# Dental implant placement with simultaneous localized ridge augmentation using L-shaped titanium mesh in the esthetic zone: a case report

**DOI:** 10.1093/jscr/rjae185

**Published:** 2024-03-27

**Authors:** Ziad Albash, Eva Hnaino, Ali Khalil

**Affiliations:** Department of Oral and Maxillofacial Surgery, Faculty of Dentistry, Tishreen University, Latakia, Syria; Faculty of Dentistry, Tishreen University, Latakia, Syria; Department of Oral and Maxillofacial Surgery, Faculty of Dentistry, Manara University, Latakia, Syria

**Keywords:** esthetic zone, guided bone regeneration, L-shaped titanium mesh, localized ridge augmentation, dental implant, case report

## Abstract

The aim of this case report is to illustrate a successful technique for dental implant placement in the esthetic zone using simultaneous localized ridge augmentation with L-shaped titanium mesh. A 35-year-old patient presented with a single missing tooth in the esthetic zone requiring dental implant placement. The treatment plan was made to place a dental implant in conjunction with a guided bone regeneration procedure using a prefabricated L-shaped titanium mesh. The procedure achieved successful reconstruction of the deficient ridge, providing ample volume and contour for implant placement. Implant osteointegration was achieved, resulting in a satisfactory functional and esthetically pleasing outcome. The use of L-shaped titanium mesh offers superior stability and biocompatibility, ensuring optimal support and containment of graft material. This case report highlights the feasibility and clinical effectiveness of dental implant placement with simultaneous localized ridge augmentation using L-shaped titanium mesh in the esthetic zone. Further studies are warranted to assess the long-term success and esthetic outcomes of this technique.

## Introduction

Dental implant placement in the esthetic zone requires special conditions for a long-term success. These conditions include adequate peri-implant bone volume, especially at buccal aspect, adequate soft tissue thickness, and thick gingival biotypes [1, 2]. Failure to strictly observe these conditions leads to functional and esthetic problems such as gingival recession, Implant Thread Exposure, and long-term failure [3, 4].

Horizontal ridge defects represent one of the toughest functional and esthetic challenges for implant placement in the esthetic zone. These defects occur due to long-term teeth loss, advanced periodontitis, or congenitally missing teeth [5, 6]. Several approaches have been suggested to augment the alveolar ridge for dental implant placement include inlay bone block grafting, bone splitting, guided bone regeneration, and distraction osteogenesis [7, 8].

During the Guided Bone Regeneration (GBR) procedure, it is necessary to use a space maintenance device to create a space for the new bone formation. Some space maintenance devices also serve as a barrier membrane such as titanium reinforced membrane and titanium mish. This is necessary to prevent the migration of nonosteogenic tissues into the area [9]. We discuss a case of dental implant placement with simultaneous localized ridge augmentation using L-shaped titanium mesh in the esthetic zone

## Case presentation

A 35-year-old female patient presented to the Oral and Maxillofacial Surgery department at university hospital with chief complaints, desire to replace the right upper canine with dental implant. Patient was recommended an Orthopantomography (OPG) and cone beam computed tomography (CBCT) for planning the implantation. OPG showed an appropriate mesiodistal distance for dental implant placement. A CBCT scan showed insufficient alveolar ridge width for dental implant placement. The average width of the alveolar ridge before the surgery was 4.56 mm. The treatment plan was made by placing a 3.5 mm diameter dental implant in conjunction with a guided bone regeneration procedure using a prefabricated L-shaped titanium mesh.

**Figure 1:**
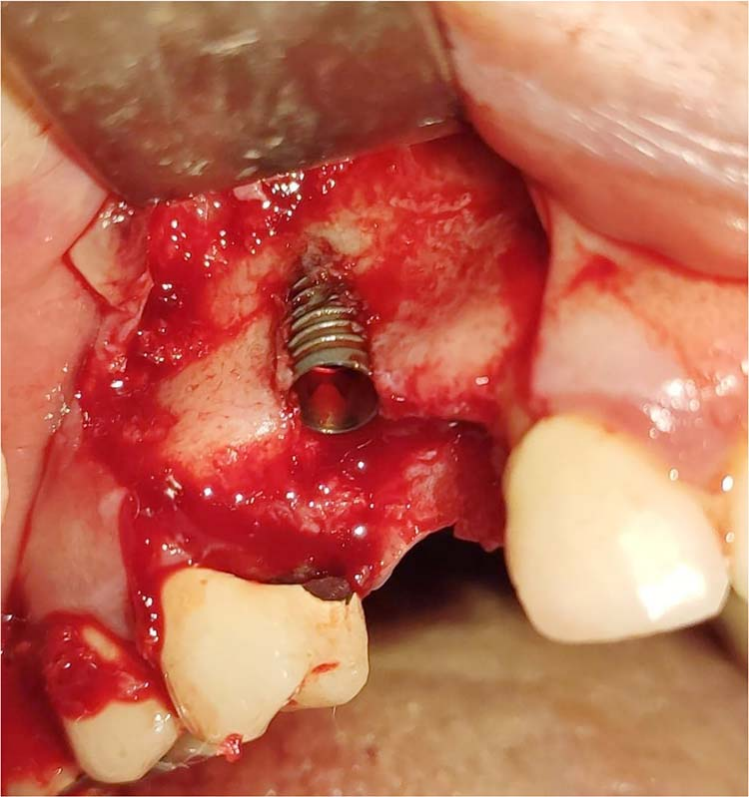
Inserting a dental implant of 3.5 mm diameter and 10 mm length into its prepared bed. The implant was completely surrounded by bone in its apical third only.

**Figure 2:**
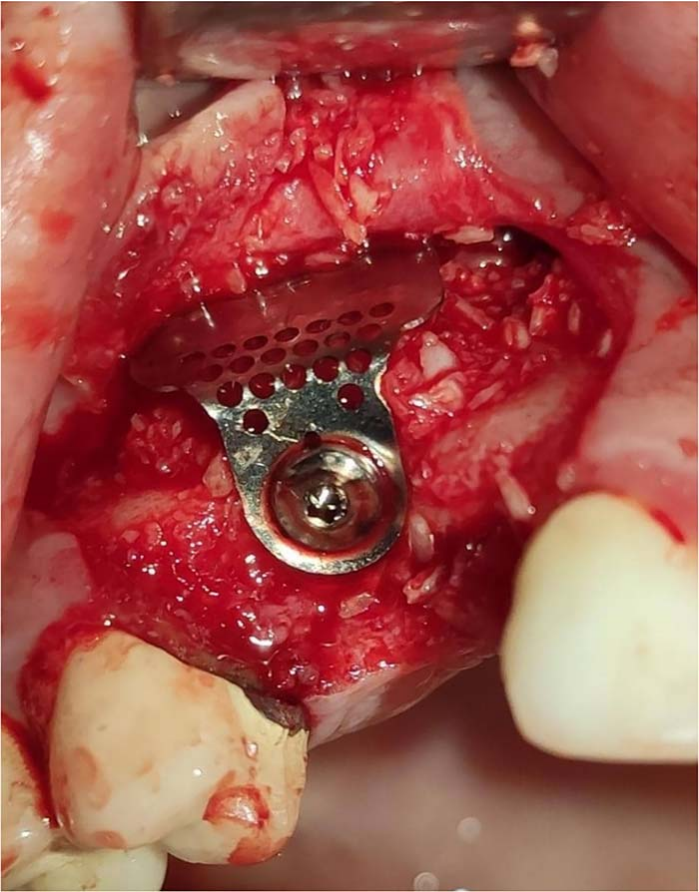
Reconstruction of the buccal plate using an allogeneic bone graft material and a prefabricated L-shaped titanium mesh. The titanium mesh was fixed to the implant with a cover screw.

Under local anesthesia with 4% articaine solution, a full-thickness flap was raised to expose the alveolar ridge. The initial point was marked with a point drill. The implant site was first prepared with a 2.2 mm pilot drill, and then with a 3.3 mm drill. After preparing the implant bed, we noticed the disappearance of the coronal and middle third of the buccal wall, exactly as planned. A submerged implant system (INNO submerged implant; Cowellmedi Inc, South Korea) was inserted according to the manufacturer’s instructions (Fig. 1). The insertion torque value was 33 N.cm. several perforations were prepared at the buccal side of the recipient bone bed using a small round bur for better blood supply. An allogeneic bone graft material (Cortical Cancellous powder; TRCIR Co, Iran) was used to reconstruct the buccal plate. A prefabricated L-shaped titanium mesh has been adapted to fit the shape of the alveolar ridge to be reconstructed, and it was fixed to the implant with a cover screw (Fig. 2). The flap was mobilized to permit a tension-free primary closure, was closed with 4–0 silk sutures. Sutures removal was done after 1 week. The surgical sites were left to heal for 6 months.

**Figure 3:**
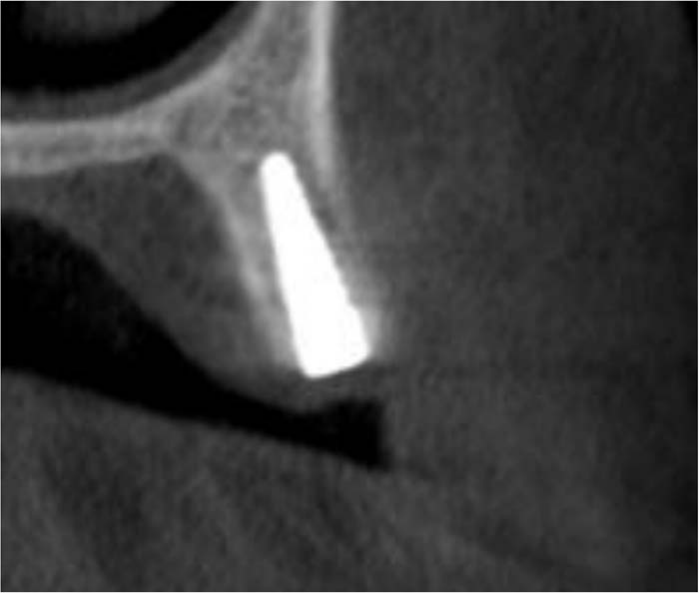
A CBCT scan 6 months after surgery showed formation of new bone buccally, and the entire dental implant being surrounded by bone.

The implant was exposed 6 months postoperatively and the healing caps were placed. CBCT scans were taken to assess gained bone width (Fig. 3). The mean alveolar ridge width before surgery was 4.56 mm. After 6 months, the mean alveolar ridge width was 7.23 mm.

## Discussion

Guided bone regeneration procedures require four crucial principles (PASS principle): primary wound closure, angiogenesis, space maintenance, and stability of bone graft materials [10]. In addition, GBR procedures require placement of a barrier that prevents the invasion of unfavorable tissue into the bone defect.

Varieties of resorbable and nonresorbable membranes have been used in GBR procedures. Resorbable membranes, such as collagen-based membranes, avoid the need for a second surgical procedure to remove the membrane, as they naturally degrade over time. These membranes stimulate soft tissue healing and enhance bone regeneration during the healing process. However, their stability and ability to contain graft material may be inferior to titanium mesh. Nonresorbable membranes, such as expanded polytetrafluoroethylene (ePTFE) membranes, have been widely used in GBR procedures [11]. ePTFE membranes provide a barrier function to prevent soft tissue invasion into the defect site, allowing undisturbed bone regeneration. They offer good stability and can be easily adapted to the defect site. However, a second surgical procedure is required to remove the nonresorbable membrane once bone regeneration is achieved. It is important to consider the specific characteristics and limitations of each membrane type when selecting the most appropriate approach for GBR procedures [12].

Titanium mesh has gained popularity for ridge augmentation procedures due to its unique properties and advantages compared with other membranes. When comparing titanium mesh with other membranes, it is important to consider factors such as stability, biocompatibility, and overall clinical outcomes. Titanium mesh offers superior stability due to its rigidity and ability to maintain its shape, providing excellent support for the augmented ridge during the healing process [13]. Additionally, the L-shaped design allows for precise adaptation to the bone defect, resulting in enhanced graft containment and stability, and allows the mesh to be fixed to the dental implant using the cover screw [13, 14]. Several studies have reported successful bone regeneration and implant osseointegration when using titanium mesh, making it a reliable option for localized ridge augmentation in the esthetic zone. Titanium mesh has shown excellent biocompatibility, minimizing adverse tissue reactions and promoting favorable osseous integration [13, 14].

Titanium mesh, with its superior stability and biocompatibility, may be particularly advantageous in cases requiring additional support and containment of graft material, such as localized ridge augmentation in the esthetic zone [13]. In this case report, we presented a successful approach for dental implant placement in the esthetic zone using simultaneous localized ridge augmentation with L-shaped titanium mesh.

Comparing the GBR techniques, namely one stage GBR and two stage GBR, both approaches have their own merits and considerations. One stage GBR involves simultaneous implant placement and ridge augmentation, minimizing the number of surgical interventions and overall treatment time. This technique offers convenience for both patients and clinicians, resulting in reduced chairside time and potential cost savings. On the other hand, two stage GBR involves a staged approach with a healing period between the ridge augmentation and implant placement. This technique allows for optimal soft tissue healing and maturation before implant placement, potentially leading to improved esthetic outcomes [13, 15]. Furthermore, it allows for a comprehensive assessment of the grafted ridge, ensuring its suitability for successful implant integration. The selection of the appropriate GBR technique depends on various factors, including the esthetic demands, bone quality, and patient-specific considerations. Clinicians must carefully evaluate each case to determine the most suitable approach.

## Conclusion

This case report highlights the feasibility and clinical effectiveness of dental implant placement with simultaneous localized ridge augmentation using L-shaped titanium mesh in the esthetic zone. Further studies are warranted to assess the long-term success and esthetic outcomes of this technique.

## Conflict of interest statement

None declared.

## Funding

None declared.

## Consent for Publication

Written informed consent was obtained from the patient for publication of this study.

## Data availability

All corresponding data used to support the findings of this study are included within the article.
